# Validation of a Clinical Prediction Rule for Distinguishing Bacterial and Aseptic Meningitis in Pediatric Patients

**DOI:** 10.7759/cureus.45829

**Published:** 2023-09-23

**Authors:** Amelia L Gurley, Matt Fukuda, Ashwin Sharma, Ethan Lee, Erica Junqueira, Michael Kang, Tommy Y Kim

**Affiliations:** 1 Emergency Medicine, Hospital Corporation of America (HCA) Healthcare, Riverside Community Hospital, Riverside, USA; 2 Emergency Medicine, University of California (UC) Riverside School of Medicine, Riverside, USA; 3 Emergency Medicine, School of Medicine, Loma Linda University, Riverside, USA; 4 Emergency Medicine, Hospital Corporation of America (HCA) Houston Healthcare, Houston, USA

**Keywords:** pediatric emergency medicine, validation study, validation, scores, pediatric meningitis

## Abstract

Introduction

The treatments and prognosis of bacterial meningitis differ greatly from those of aseptic meningitis, making early identification and differentiation essential. Several different clinical prediction rules have been developed to distinguish bacterial meningitis from aseptic meningitis. We sought to validate one clinical prediction rule for pediatric patients utilizing a centralized data warehouse that collects daily data from 184 hospitals across the United States.

Methods

We retrospectively collected data on all patients aged 29 days to 14 years who presented to Hospital Corporation of America (HCA) Healthcare hospitals from January 1, 2016, to May 31, 2021, with a diagnosis of meningitis. Our study replicated the original study of the meningitis score for emergencies (MSE) for the pediatric clinical prediction rule and assigned 3 points for procalcitonin (PCT) >1.2 ng/dL, 2 points for CSF protein >80 mg/dL, and 1 point for each of the other variables of C-reactive protein (CRP) >40 mg/L and CSF absolute neutrophil count >1000 cells per mm^3^. Patients were categorized either as having bacterial or aseptic meningitis. Using the clinical prediction rule, a calculation of the sensitivity, specificity, positive predictive value, negative predictive value, and receiver operating characteristic (ROC) curve was performed.

Results

The optimum test characteristic was found to have a score of ≥ 3, showing a sensitivity of 92.86% (95% CI, 83.3-100), a specificity of 65.22% (95% CI, 51.5-79), a positive predictive value of 61.90% (95% CI, 47.2-76.6), and a negative predictive value of 93.75% (95% CI, 85.4-100). The ROC curve from this study showed an area under the curve (AUC) of 0.7892 (95% CI, 0.681-0.897).

Conclusion

Our study validated a high sensitivity for distinguishing bacterial meningitis from aseptic meningitis, suggesting the clinical prediction rule has clinical utility as a predictive screening tool. Although the original MSE advised a cutoff score of ≥1, our study suggests that a score ≥3 would give the best test performance.

## Introduction

Early identification of bacterial vs. aseptic meningitis is essential, as the treatments and prognoses of these conditions differ greatly [1,2]. Aseptic meningitis is estimated to be responsible for 26,000 to 42,000 hospitalizations annually in the US [3]. Kaur et al. noted that these numbers are likely low due to underreporting [1]. Furthermore, evidence suggests that rates of aseptic meningitis are higher in pediatric and specifically infant populations. A study of aseptic meningitis in South Korea found a higher incidence in children aged less than one year and from four to seven years [4]. Mount and Boyle noted that rates of aseptic meningitis in Europe were 10 times higher in children under one year of age compared to adult populations [2]. This may be because several causes of aseptic meningitis are more often seen in pediatric populations, including but not limited to enterovirus infection, vaccine administration, many types of neoplasm, and initial exposure to medications such as non-steroidal anti-inflammatory drugs (NSAIDs) or penicillins that can lead to drug-induced aseptic meningitis. The higher relative rate of aseptic meningitis compared to bacterial meningitis in children may also be due to decreasing rates of the latter in young children after the advent of vaccines effective against *Haemophilus influenzae* type b and *Streptococcus pneumoniae*, two of the most common historical sources of bacterial meningitis in young pediatric populations.

Furthermore, both clinical symptoms and laboratory signs of aseptic and bacterial meningitis vary in pediatric and adult populations. Many studies have noted that children are less likely to present with classic meningeal complaints of stiff necks, nausea, or photophobia [1,3,5,6]. Cerebrospinal fluid gram stain findings alone have been repeatedly found insufficient to accurately diagnose bacterial meningitis in infants [5,7,8].

In September 2020, Mintegi et al. published an article titled *Clinical Prediction Rule for Distinguishing Bacterial from Aseptic Meningitis *[9]. In this study of 1007 pediatric patients aged 29 days to 14 years of age, they derived a meningitis score for emergencies (MSE) to distinguish bacterial from aseptic meningitis [9]. Assigning 3 points for procalcitonin (PCT) >1.2 ng/dL, 2 points for CSF protein >80 mg/dL, 1 point for CSF absolute neutrophil count >1000 cells per mm3, and 1 point for C-reactive protein (CRP) >40 mg/L, they were able to calculate an MSE ranging from 0 to 7 [9]. They found an MSE of ≥1 predicted bacterial meningitis with a sensitivity of 100% (95% CI, 95-100), a specificity of 83.2% (95% CI, 80.6-85.5), and a negative predictive value of 100% (95% CI, 99.4-100) [9]. The gold standard of aseptic meningitis was defined in this paper by the absence of bacterial strains on CSF culture, blood culture, and polymerase chain reaction (PCR) identification of the bacterial strains *Neisseria meningitidis* or *S. pneumoniae*. Bacterial meningitis was defined in this paper by either a positive CSF culture of bacteria, by positive CSF PCR identification of *N. meningitidis* or *S. pneumonia*e, or by CSF pleocytosis (≥10 WBCs per mm3) in the setting of a positive blood culture or PCR [9].

We performed a retrospective validation study of the pediatric MSE by obtaining data from the Hospital Corporation of America (HCA) Healthcare centralized data warehouse. Our primary hypothesis was that the MSE would accurately distinguish bacterial from aseptic meningitis in a different cohort of children from the original study group.

## Materials and methods

Study design

Our study replicated the original study by Mintegi et al. Data was obtained from the HCA Healthcare data warehouse by a data analyst. We retrospectively collected data on all patients aged 29 days to 14 years who presented to HCA Healthcare hospitals from January 1, 2016, to May 31, 2021, with a diagnosis of meningitis. We included patients with complete laboratory values of a PCT, CSF protein, CSF neutrophil count, CRP, CSF culture and/or CSF PCR tests, and blood culture and/or blood PCR tests. We excluded any patient without the complete laboratory values stated above or any patient with immunosuppression, the presence of a mechanical device (indwelling catheter, ventriculoperitoneal shunt, auditory prosthesis), or a CSF fistula. Patient data was obtained from the HCA Healthcare Data Warehouse, a centralized data warehouse that collects daily data from 184 hospitals, mainly community and non-academic centers, in 20 states across the United States. This study was granted an exemption by the Institutional Review Board (IRB) of our institution.

Using the following variables, we used the MSE's original scoring system to assign 3 points for PCT >1.2 ng/ml, 2 points for CSF protein >80 mg/dL, and 1 point for each of the other variables of CSF absolute neutrophil count >1000 cells per mm3 and CRP >40 mg/L. Patients were categorized either as having bacterial or aseptic meningitis. Using the MSE, a calculation of the sensitivity, specificity, positive predictive value, and negative predictive value and the generation of a receiver operating characteristic (ROC) curve were performed by an HCA Healthcare statistician.

Definitions

Bacterial Meningitis

Patients with bacterial meningitis were defined as having either one of the following two criteria: (1) identification of a bacterial pathogen in CSF by growth in bacterial culture and/or genomic detection of bacteria in CSF using PCR testing; or (2) presence of CSF pleocytosis (10 WBCs per mm3) and either a positive blood culture result and/or genomic detection of bacteria in blood using PCR testing. Certain bacterial species (including *Staphlyococcus epidermidis*, *Propionibacterium acnes*, *Streptococcus viridans*, *Corynebacterium spp.*, and other diphtheroids) isolated in otherwise healthy patients were considered contaminants.

Aseptic Meningitis

The category of aseptic meningitis was defined as patients with CSF pleocytosis and negative CSF and blood bacterial cultures, as well as negative genomic testing using PCR tests.

Pleocytosis 

Pleocytosis was defined as having CSF WBCs of greater than 10 cells per mm3, corrected for the presence of CSF RBCs by using a 1:500 ratio of leukocytes to erythrocytes usually found in peripheral blood [10]. The CSF protein was also corrected by increasing CSF protein by 1.1 mg/dL for every 1000-cell increase in CSF RBC per mm3 [11].

## Results

Our final sample size included 74 patients, with 28 (37.8%) diagnosed with bacterial meningitis (Table 1). Our inclusion criteria resulted in 237 admissions. Twenty-six were excluded due to a contaminated CSF culture, and two were excluded as readmissions for the same patient. One hundred thirty-two were excluded due to incomplete laboratory testing, and three were excluded due to no cultures being performed. Patients with bacterial meningitis were younger in age compared to those with aseptic meningitis (14.9 months vs. 25.0 months, p = 0.30). More female patients had bacterial meningitis compared to aseptic meningitis (46.4% vs. 30.4%, p = 0.16).

**Table 1:** List of organisms in the bacterial meningitis group * 8 patients with multiple organisms: 1st patient with *Streptococcus agalactiae,* and *Enterobacter cloacae;* 2nd patient with *Haemophilus influenza*, *Klebsiella pneumonia*, and *Streptococcus pneumoniae;* 3rd patient with *K. pneumoniae*, and *Staphylococcus aureus;* 4th patient with *Escherichia coli*, *Serratia marcescens*, and *Enterococcus faecalis;* 5th patient with *S. agalactiae*, and *K. pneumoniae; *6th patient with *E. coli*, and *S. pneumoniae;* 7th patient with *S. Aureus*, and *Enterobacter aerogenes*; 8th patient with *S. pneumoniae*, and *Candida albicans*

Organism	Total number of patients
Streptococcus agalactiae	6
Streptococcus pneumoniae	6
Escherichia coli	2
Haemophilus influenzae	2
Stenotrophomonas maltophilia	1
Streptococcus pyogenes	1
*Salmonella *species	1
Streptococcus galloyticus	1
Multiple organisms*	8

Using Mintegi et al.'s cut-off of an MSE of ≥1, our results predicted bacterial meningitis with a sensitivity of 92.86% (95% CI, 83.3-100), a specificity of 30.43% (95% CI, 17.1-43.7), a positive predictive value of 44.80% (95% CI, 32.0-57.6), and a negative predictive value of 87.50% (95% CI, 71.3-100). Our optimum test characteristic was found with an MSE of ≥3, showing a sensitivity of 92.86% (95% CI, 83.3-100), a specificity of 65.22% (95% CI, 51.5-79), a positive predictive value of 61.90% (95% CI, 47.2-76.6), and a negative predictive value of 93.75% (95% CI, 85.4-100). The ROC curve from this study was well above the chance diagonal line, and the area under the curve (AUC) was 0.7892 (95% CI, 0.681-0.897).

## Discussion

There have been multiple predictive scoring tools developed to allow clinicians to exclude bacterial meningitis, thereby pursuing less aggressive treatments. The MSE and the bacterial meningitis score (BMS) are the most well-known. The BMS was originally validated in a single-site retrospective study of 699 patients and was found to have a negative predictive value of 100% (95% CI, 97-100) and sensitivity of 87% (95% CI, 72-96) [12]. It has since been validated by multiple independent studies [13-15]. A 2012 meta-analysis used all studies published between 2002 and 2012 with sufficient data to calculate the BMS (eight studies comprising 4896 patients) and found a combined sensitivity of 99.3% (95% CI, 98.7-99.7), specificity of 62.1% (95% CI 60.5-63.7), negative predictive value of 99.7% (95% CI 99.3-99.9), positive likelihood ratio of 2.6 (95% CI 2.5-2.7), and negative likelihood ratio of 0.01 (95% CI 0.01-0.02) [15]. One 2006 study noted that the BMS “appears to be the only one that offers 100% sensitivity, good specificity (66% for our population and 73% with the author's validation set), and greater ease of manual computation at the bedside” [16]. Such high sensitivities suggest the BMS can serve as a useful way to identify patients who would not benefit from early antibiotic therapy and hospitalization.

However, the BMS was found to specifically misidentify infants as being at lower risk, making this screening tool much more dangerous to use in young pediatric populations [12,13,15]. Multiple additional tools have since been developed to address this problem, including not only the MSE but also models devised by Freedman et al., Nigrovic et al., Bonsu and Harper, Bonsu et al., and Oostenbrink et al. [8,14,16,17,18]. In 2023, after this study had begun, Wu et al. published yet another screening tool designed specifically for patients aged 29 to 90 days [19]. Unfortunately, many of these models have not yet been validated externally or have since failed external validation [14,16,19,20]. Further study is needed to evaluate screening tools’ utility in assessing bacterial vs. aseptic meningitis in pediatric patient populations and to identify a reliable tool for use in infant populations.

Our results, like Mintegi et al.'s original study, found that the MSE has a higher sensitivity than the BMS among pediatric populations, which suggests that the MSE may be more useful for pediatric patients [9,12,13]. Our results do suggest a poorer predictive value for an MSE of ≥1 across every predictive parameter when compared to the original study by Mintegi et al. We found a sensitivity of 92.86% compared to 100%, a specificity of 30.4% compared to 83.2%, and a negative predictive value of 87.5% compared to 100%, although there was overlap within each confidence interval (Table 2). This implies that, while there is useful negative predictive value in the MSE, an MSE of ≥1 may not be reliable for identifying pediatric bacterial meningitis. We found an MSE of ≥3 to have a better ability to rule in or identify cases of bacterial meningitis.

**Table 2:** Comparison of predictive value of meningitis screening tools MSE: Meningitis score for emergencies

MSE comparison	Sensitivity	Specificity	Negative predictive value	Validation
MSE ≥ 1 (Mintegi et al.)	100% (95% CI 95-100)	83.2% (95% CI 80.6-85.5)	100% (95% CI 99.4-100)	Single-site retrospective study of 1007 patients aged 29 days to 14 years
MSE ≥ 1 (our results)	92.9% (95% CI 83.3-100)	30.4% (95% CI 17.1-43.7)	87.5% (95% CI 71.3-100)	Single nation, multi-site retrospective study of 74 patients aged 29 days to 14 years
MSE ≥ 3 (our results)	92.9% (95% CI 83.3-100)	65.2% (95% CI 51.5-79)	93.75% (95% CI 85.4-100)	Single nation, multi-site retrospective study of 74 patients aged 29 days to 14 years

However, even an MSE of ≥3 would, at the lowest end of the confidence interval, potentially miss bacterial meningitis in up to 3 out of every 20 potential cases, according to our results. Like any other predictive screening tool, the MSE was not intended to be used in isolation. An MSE score alone is no substitute for CSF cultures, which remain the gold standard for diagnosis. Our study does suggest, however, that an MSE of ≥3 may have utility as a screening tool to reduce antibiotic use in pediatric patients.

Limitations of our study include a small sample size. We included 74 patients, compared to 1009 included in Mintegi et al.'s original study, which could affect the difference in the results during our validation [9]. We also did not exclude patients based on the timing of antibiotic administration, as this data is not recorded, so it is possible that there were patients who received antibiotics prior to culture, causing falsely negative culture results. As a retrospective cohort study, our study specifically examined the utility of the MSE in identifying confirmed cases of bacterial meningitis, not the MSE's ability to predict aseptic meningitis. However, our study's small size and retrospective design do not change the results regarding sensitivity or specificity. We found a high sensitivity and negative predictive value, which suggests the MSE may be useful for ruling out patients.

Finally, while our findings regarding sensitivity suggest the MSE has utility, positive and negative predictive values are highly dependent on disease prevalence. Our study specifically examined cases of pediatric meningitis at multiple HCA Healthcare hospitals across the United States during a five-year period from 2016 to 2021, while the original MSE was validated using data from a single center over six years [9]. The geographic distribution of various bacterial pathogens and the changing incidence of bacterial and aseptic meningitis in pediatric populations over time mean that the predictive value of screening tools such as the MSE will likely continue to change when applied to different populations. It is therefore essential to continue reexamining the validity of such scoring tools with regard to different populations as time goes by.

## Conclusions

This retrospective study validated the presence of an association between a positive MSE and the presence of bacterial meningitis, suggesting the MSE has clinical utility as a predictive screening tool. However, at an MSE ≥1, our validation showed poorer predictive results than the original study with regard to specificity. This study suggests that an MSE ≥3 would give the best test performance.
